# Efficient Keyset Design for Neural Networks Using Homomorphic Encryption †

**DOI:** 10.3390/s25144320

**Published:** 2025-07-10

**Authors:** Youyeon Joo, Seungjin Ha, Hyunyoung Oh, Yunheung Paek

**Affiliations:** 1 Department of Electrical and Computer Engineering & ISRC, Seoul National University, Seoul 08826, Republic of Korea; yyjoo@sor.snu.ac.kr (Y.J.); sjha@sor.snu.ac.kr (S.H.); 2 Department of AI, Gachon University, Seongnam-si 13120, Republic of Korea

**Keywords:** machine learning as a service, privacy-preserving machine learning, neural networks, fully homomorphic encryption, CKKS, rotation keyset, cryptography, privacy-preserving techniques, intrusion detection system

## Abstract

With the advent of the Internet of Things (IoT), large volumes of sensitive data are produced from IoT devices, driving the adoption of Machine Learning as a Service (MLaaS) to overcome their limited computational resources. However, as privacy concerns in MLaaS grow, the demand for Privacy-Preserving Machine Learning (PPML) has increased. Fully Homomorphic Encryption (FHE) offers a promising solution by enabling computations on encrypted data without exposing the raw data. However, FHE-based neural network inference suffers from substantial overhead due to expensive primitive operations, such as ciphertext rotation and bootstrapping. While previous research has primarily focused on optimizing the efficiency of these computations, our work takes a different approach by concentrating on the rotation keyset design, a pre-generated data structure prepared before execution. We systematically explore three key design spaces (KDS) that influence rotation keyset design and propose an optimized keyset that reduces both computational overhead and memory consumption. To demonstrate the effectiveness of our new KDS design, we present two case studies that achieve up to 11.29× memory reduction and 1.67–2.55× speedup, highlighting the benefits of our optimized keyset.

## 1. Introduction

Smart devices and sensors are now used in various fields such as the Internet of Things (IoT). Being very close to our daily lives, they generate vast amounts of data and process the data to provide meaningful results for diverse applications. For instance, wearable devices detect human behavior and collect privacy-sensitive health-related data, such as electrocardiograms (ECGs) [1,2] and blood pressure [3,4,5].

Driven by advances in AI, the analysis of such data has continually developed and evolved. Table 1 presents prior works demonstrating AI models trained on datasets that could be acquired through various sensors embedded in wearable or mobile devices. In general, the larger the AI model, the higher the quality (accuracy) of the AI service [6,7,8]. Such large models require larger amounts of memory to store codes, parameters, and hyperparameters. However, conventional smart devices typically have limited resources, which makes it infeasible to implement and run the full inference computation model on them [9,10].

To address such concerns, AI service providers typically perform inference tasks on remote servers, a paradigm known as Machine Learning as a Service (MLaaS). Specifically, the user sends a query containing input data to the server, which performs inference and returns the result to the user’s device. However, this practice raises another issue related to the privacy of the users’ sensitive personal data [41,42,43,44,45]. A somewhat contradictory characteristic of modern AI services is that, as they become more deeply integrated into our daily lives, they increasingly rely on highly sensitive and private user data as input. Although such data are typically transmitted in encrypted form, they are often processed in raw and unencrypted form at the remote server. This creates a serious risk—unauthorized access or data leakage during processing can result in serious privacy violations, raising significant ethical and security concerns [46,47,48,49,50]. Although regulations such as the General Data Protection Regulation (GDPR) have been enacted to address privacy issues, they do not alter the following fundamental limitation: the need to decrypt data during processing.

**Figure 1:**
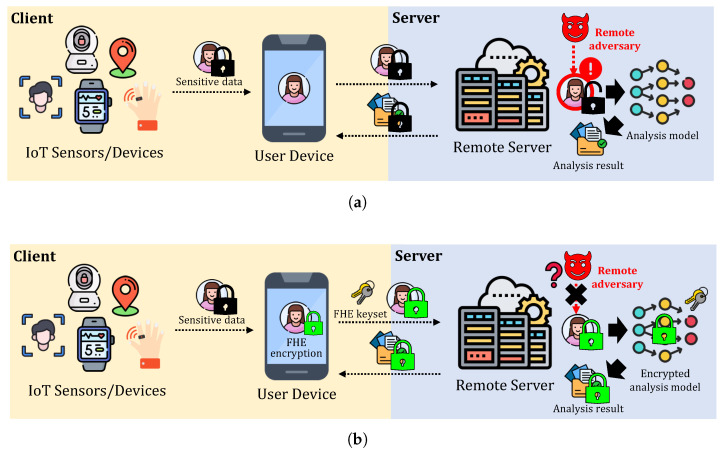
Comparison of two-party MLaaS to a remote server using data from IoT sensors/devices. The black lock represents data encryption using conventional encryption schemes (e.g., AES), while the green lock represents encryption with the FHE. (**a**): MLaaS with conventional encryption, where both sensitive data and analysis results are exposed to the server due to the lack of support for computations on encrypted data. (**b**): MLaaS with FHE, where sensitive data remains protected throughout the entire analysis process. Note that the transmitted FHE rotation keyset does not include a secret key.

Privacy-Preserving Machine Learning (PPML) has emerged as a critical research area to address these challenges, aiming to enable secure model inference while preventing the exposure of sensitive data. Among the various cryptographic techniques proposed for PPML, Fully Homomorphic Encryption (FHE) is one of the promising privacy-preserving techniques that is widely researched for PPML. FHE allows computations to be performed directly on encrypted data, ensuring that the sensitive information remains secure throughout the entire computation process. Figure 1 illustrates the examples of MLaaS with and without FHE, where FHE-encrypted MLaaS ensures data protection throughout the entire remote inference, including results, while the other is vulnerable to the exposure of sensitive data. However, this enhanced security comes at the cost of increased computational overhead. FHE ciphertexts are typically represented as high-dimensional polynomial structures, leading to significant memory and computational requirements. In addition to basic arithmetic operations such as addition and multiplication, FHE computations involve expensive primitives, including relinearization, rotation, and bootstrapping, all of which contribute to considerable performance degradation compared to unencrypted computations. As a result, improving the efficiency of FHE-based machine learning has been a key challenge in the deployment of practical PPML solutions.

While prior works [51,52,53,54,55] have significantly contributed to improving FHE efficiency, the configuration of the *FHE keyset* directly affects both performance and memory consumption but has received little attention in prior research. This paper presents a comprehensive analysis of the FHE keyset design space (KDS) and introduces novel methodologies to optimize keysets tailored to specific applications. An FHE keyset typically consists of public keys, relinearization keys, and rotation keys, and it also includes bootstrapping keys if bootstrapping is used. These keys are pre-generated on the client side and transmitted to the server before execution, where key assignment can be tailored dynamically. For instance, a rotation by index 7 can be computed either directly by using the rotation key for index 7 or indirectly by decomposing 7 into 4 + 2 + 1 and applying three separate rotations using the corresponding keys. Despite its significance, existing studies have largely treated the keyset as a fixed control variable rather than an optimizable component. In contrast, our work identifies the keyset’s design as a manipulable variable that can be systematically optimized to improve both execution speed and memory efficiency.

Considering the keyset design as a manipulative variable, we observe that the keyset can be designed with three parameters: rotation index, ciphertext level, and the number of temporal primes, *α* (we provide details of their specifications in the following sections). Although some prior work has suggested the possibility of modified keysets, they typically consider only one or two of these three factors. Moreover, they lack application awareness, focusing only on shallow analyses involving single multiplications or rotations without considering end-to-end inference workloads. A key novelty of our work is the introduction of a path-finding methodology for discovering optimal keysets tweaked for a specific neural network architecture. Our exploration of the keyset design space (KDS) encompasses all three parameters in a comprehensive way. To demonstrate the practical utility of our approach, we consider the well-known PPML as an application, designing different keysets for different neural networks. Based on our experiments, we propose several keyset designs that are different from the customary ones, resulting in better performance or memory consumption. The results suggest that our keyset design approach achieves 1.67–2.55× faster inference latency compared to previous approaches, reducing memory consumption up to 11.29×.

The remainder of this paper is organized as follows. Section 2 provides the essential preliminaries for understanding this work. Section 3 presents two key motivations behind our work, explaining the rationale for this work. Then, in Section 5, we introduce three factors of the keyset design space in detail and propose our approach, which aims to minimize both latency and memory consumption. The experimental results are presented in Section 6, while Section 4 discusses related research, and Section 7 covers additional aspects and the broader implications of our work. Finally, Section 8 concludes our paper.

## 2. Background

This section presents the essential theoretical background of the RNS-CKKS scheme that will appear throughout our study. For readers who are interested and are seeking more details about RNS-CKKS or FHE, we leave references with more elaborate explanations [56,57] about them.

### 2.1. RNS-CKKS

The CKKS scheme [56,57] is a homomorphic encryption scheme designed for computation over real and complex numbers, allowing arithmetic operations to be performed directly on encrypted data. It encodes vectors of real or complex numbers into plaintext polynomials by the inverse of the canonical embedding $\tau^{-1}$ with a scaling factor Δ, which returns $\left\lceil \Delta \cdot \tau^{-1}\left(m\right)\right\rfloor$ for a given vector $m\in C^{\frac{N}{2}}$. The plaintext space is defined as a polynomial ring $R_{Q,N}=Z_{Q}\left[X\right]/\left(X^{N}+1\right)$, where *N* is a power of two (N≥2), determining the degree of the polynomial, and *Q* is a ciphertext modulus that influences both precision and security. To improve computational efficiency, RNS-CKKS utilizes the Residue Number System (RNS). In RNS-CKKS, the ciphertext modulus *Q* is decomposed into a product of smaller primes based on the Chinese Remainder Theorem (CRT), which is defined as $Q=\prod_{i=0}^{k}q_{i}$. This decomposition enables parallel computation and eliminates expensive large-integer arithmetic. RNS-CKKS supports arithmetic operations directly on ciphertexts, including addition and multiplication. The addition is performed element-wise between two ciphertexts ${\mathrm{ctx}}_{1}=\left(c_{0}^{\left(1\right)},c_{1}^{\left(1\right)}\right)$ and ${\mathrm{ctx}}_{2}=\left(c_{0}^{\left(2\right)},c_{1}^{\left(2\right)}\right)$, and their sum is computed as ${\mathrm{ctx}}_{\mathrm{add}}=\left(c_{0}^{\left(1\right)}+c_{0}^{\left(2\right)},c_{1}^{\left(1\right)}+c_{1}^{\left(2\right)}\right)$. The addition does not significantly increase noise, making it inexpensive. Multiplication is also element-wise, for given two ciphertexts ${\mathrm{ctx}}_{1}$ and ${\mathrm{ctx}}_{2}$, their product is defined as ${\mathrm{ctx}}_{\mathrm{mult}}={\mathrm{ctx}}_{1}\cdot {\mathrm{ctx}}_{2}$. The other types of operation RNS-CKKS supports are rescaling and rotation. Unlike addition, multiplication increases noise and doubles the scale factor ($\Delta^{2}$), requiring rescaling to restore the scale factor (Δ). Rescaling prevents excessive scale growth by dividing the ciphertext coefficients by a prime modulus, $\mathrm{Rescale}\left(\mathrm{ctx}\right)=\left\lfloor \frac{\mathrm{ctx}}{q_{i}}\right\rfloor$. Rescaling ensures numerical stability and prevents overflow. Rotation is another crucial operation that requires a special pre-computed rotation key, which will be detailed in the following sections.

### 2.2. Ciphertext Level and Bootstrapping

As previously mentioned, the ciphertext modulus is expressed as $Q=\prod_{i=0}^{l}q_{i}$, where $q_{0}$ is the base modulus, and $q_{1},\ldots ,q_{l}$ denotes the prime moduli forming the RNS representation [57]. Here, the parameter *l* represents the ciphertext level, which indicates how many homomorphic multiplications can still be performed. When homomorphic multiplications are performed, the ciphertext level must be reduced to keep the ciphertext scale constant as Δ. This process, known as rescale, reduces the ciphertext modulus *Q* by removing its last prime factor. After each multiplication, the ciphertext modulus is updated as $Q^{\prime }=\frac{Q}{q_{l}}$. Since ciphertexts in RNS-CKKS have a fixed scaling factor $\Delta \approx q_{l}$, rescaling reduces the scale by dividing by Δ, $Q^{\prime }=\frac{Q}{\Delta }$, and the ciphertext level is reduced from *l* to l−1.

At the lowest level of a ciphertext, for more multiplications, it should recover the exhausted levels. Bootstrapping [51,58] enables this recovery by performing homomorphic operations in four steps: slotToCoeff, modRaise, modReduction, and coeffToSlot. Bootstrapping begins with slotToCoeff at the lowest level of a ciphertext, which homomorphically performs CKKS decoding operations (i.e., evaluation of the DFT matrix), including several rotations and multiplications. When modRaise raises the ciphertext modulus, the encrypted space is converted, so the message cannot be decrypted correctly. Thus, homomorphic modular reduction is evaluated to achieve a properly encrypted message. Finally, coeffToSlot homomorphically performs CKKS encoding (i.e., evaluation of the iDFT matrix), including rotations and multiplications.

### 2.3. Key Switching

Key switching is a fundamental operation in RNS-CKKS that transforms ciphertexts into an equivalent form under a different secret key. This process is necessary for rotation operations to enable ciphertext slot manipulations and for linearization operations to ensure that the ciphertext remains decryptable after multiplication under the same secret key. Key switching allows a ciphertext encrypted under a secret key $s_{1}$ to be transformed into an equivalent ciphertext under a secret key $s_{2}$, ensuring continued compatibility in homomorphic computations. For example, after multiplication between two ciphertexts, the result dimension increases $\left(d_{0},d_{1},d_{2}\right)$, where the $d_{2}$ term is under the squared secret key $s^{2}$. At this time, we need to switch the key $s^{2}$ to *s* for correct decryption, which can be achieved by key switching.

Let a ciphertext at level *l* consist of two polynomials $\mathrm{ctx}=\left(c_{0},c_{1}\right)\in R_{Q_{l},N}^{2}$. The decryption under a secret key $s_{1}$ is performed as $c_{0}+c_{1}\cdot s_{1}\approx m$. Key switching transforms the ciphertext such that it can be decrypted under $s_{2}$, preserving correctness: $c_{0}^{\prime }+c_{1}^{\prime }\cdot s_{2}\approx m$. To accomplish this transformation, the key-switching procedure expresses the ciphertext in decomposed form and applies key-switching keys. Key switching relies on digit decomposition, breaking down the second polynomial component, $c_{1}=\sum_{i=0}^{\mathrm{dnum}-1}c_{1,i}\cdot B^{i}$, where dnum is the number of decomposition digits, and *B* is the decomposition base. The final transformed ciphertext is computed as ${\mathrm{ctx}}^{\prime }=\left(c_{0}+\sum_{i=0}^{\mathrm{dnum}-1}c_{1,i}\cdot K_{\mathrm{KS},i}^{\left(l\right)},0\right)$, where $K_{\mathrm{KS},i}^{\left(l\right)}$ is the pre-computed key-switching key for level *l*.

**Figure 2:**
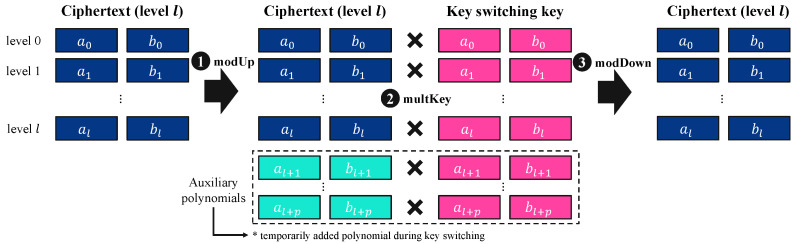
Overview of key-switching process.

Figure 2 illustrates the key-switching process, which consists of three main steps: modUp, multKey, and modDown. For a given ciphertext ctx at level *l*, the initial modulus is $Q=\prod_{i=0}^{l}q_{i}$, and modUp expands the modulus to $PQ=p\cdot \prod_{i=0}^{l}q_{i}$. This ensures that the ciphertext and the key-switching key are compatible for the upcoming transformation. The modulus-raised ciphertext ${\mathrm{ctx}}^{\prime }$ is now expressed as ${\mathrm{ctx}}^{\prime }=\left({\mathrm{ctx}\prime }^{\left(0\right)},{\mathrm{ctx}\prime }^{\left(1\right)},\ldots ,{\mathrm{ctx}\prime }^{\left(1+l\right)}\right)$, where each component corresponds to a different level. Then, in multKey, the ciphertext components are multiplied by the precomputed key-switching key $K_{\mathrm{KS}}$, enabling the secret key transition. Each component of the modulus-raised ciphertext undergoes the transformation as ${\mathrm{ctx}\prime }^{\left(0\right)}={\mathrm{ctx}\prime }^{\left(0\right)}\cdot K_{\mathrm{KS}}^{\left(0\right)}\;\mathrm{mod}\;p$ and ${\mathrm{ctx}\prime }^{\left(i\right)}={\mathrm{ctx}\prime }^{\left(i\right)}\cdot K_{\mathrm{KS}}^{\left(i\right)}\;\mathrm{mod}\;q_{i},\;\;\;\mathrm{for}\;1\leq i\leq l$. This step effectively transforms the ciphertext to be decryptable under a different secret key. After the key-switching transformation, through modDown, the ciphertext modulus is reduced back to $Q=\prod_{i=0}^{l}q_{i}$. This step ensures that the ciphertext returns to its original modulus level and that the secret key is successfully switched to the new secret key. At the end of this process, the transformed ciphertext remains valid for decryption under the new secret key.

### 2.4. Rotation

Rotation enables the slot-wise manipulations of encrypted vectors, which is crucial for matrix–vector multiplications and convolutional operations in deep learning. Since RNS-CKKS batches multiple plaintext values into a single ciphertext, an efficient rotation mechanism is required. Rotation in RNS-CKKS operates at different ciphertext levels and requires specific rotation indices to determine how far the ciphertext slots should be shifted. Since ciphertexts decrease in modulus over multiplication, rotation must be performed at the correct level. Each level *l* requires a corresponding rotation key. The rotation index *r* determines how far the slots are shifted. A ciphertext encoding $\left[m_{1},m_{2},\ldots ,m_{N}\right]$ rotated by *r* positions results in $\mathrm{Rotate}\left(\mathrm{ctx},r\right)\Rightarrow \left[m_{r+1},\ldots ,m_{N},m_{1},\ldots ,m_{r}\right]$. Since storing keys for all rotation indices is impractical, widely used FHE libraries [59,60] only precompute power-of-two rotation keys (e.g., 1, 2, 4, 8, *…*), computing arbitrary rotations recursively. Rotation is performed using a Galois automorphism $\theta_{g}\left(X\right)$, $\theta_{g}\left(X\right)=X^{g}\;\mathrm{mod}\;\left(X^{N}+1\right)$, where *g* is derived from the rotation index *r*. Since applying $\theta_{g}$ alters the ciphertext’s structure, key switching is needed. The modified ciphertext is ${\mathrm{ctx}}^{\prime }=\left(\theta_{g}\left(c_{0}\right),\theta_{g}\left(c_{1}\right)\right)$. Since $\theta_{g}\left(c_{1}\right)$ is not encrypted with the correct key, it is decomposed and key-switched, $\theta_{g}\left(c_{1}\right)=\sum_{i=0}^{\mathrm{dnum}-1}c_{1,i}\cdot B^{i}$. The final rotated ciphertext is ${\mathrm{ctx}}_{\mathrm{rot}}=\left(\theta_{g}\left(c_{0}\right)+\sum_{i=0}^{d_{\mathrm{num}}-1}c_{1,i}\cdot {\mathrm{RotK}}_{i}^{\left(l\right)},0\right)$, where ${\mathrm{RotK}}_{r}^{\left(l\right)}$ is the rotation key at level *l*.

### 2.5. FHE Keyset Structure and Generation

**Figure 3:**
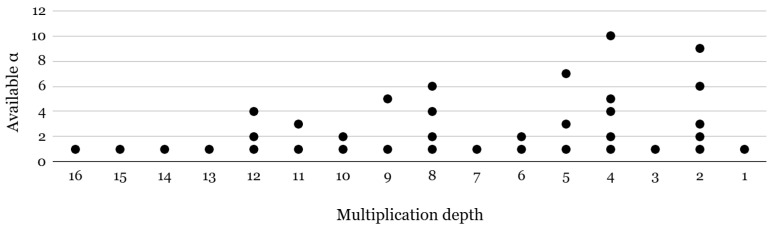
Available α options per multiplication depth for 128-bit security.

The *FHE keyset* in RNS-CKKS consists of multiple precomputed keys that facilitate key switching for relinearization and rotation. The keyset includes relinearization keys (RK), which are used to reduce the ciphertext’s size after ciphertext multiplication, and rotation keys (${\mathrm{RotK}}_{r}$), which enable homomorphic slot shifting by *r* for different indices. Since both keys rely on key switching, FHE keyset generation is based on the same key-switching key-generation algorithm $\mathrm{SWKGen}(s_{1},s_{2})$, where $s_{1},s_{2}\in R$. $\mathrm{SWKGen}(s_{1},s_{2})$ generates the switching keys:

(1) $$\left({\mathrm{SWK}}^{\left(0\right)}=\left(b^{\prime (0)},a^{\prime (0)}\right),\ldots ,{\mathrm{SWK}}^{(\alpha +l)=}=\left(b^{\prime (\alpha +l)},a^{\prime (\alpha +l)}\right)\right)\in \prod_{i=0}^{\alpha -1}R_{p_{i}}^{2}\times \prod_{j=0}^{l}R_{q_{j}}^{2}$$

where $b^{\prime (i)}↤-a^{\prime (i)}\cdot s_{2}+e^{\prime }$ (mod $p_{i}$) for 0≤i<α and $b^{\prime (\alpha +j)}↤-a^{\prime (j)}\cdot s_{2}+{\left[P\right]}_{q_{j}}+e^{\prime }$ (mod $q_{j}$) for 0≤j<l by randomly sampling $\left(a^{\prime (0)},\ldots ,a^{\prime (\alpha +l)}\right)\in \prod_{i=0}^{\alpha -1}R_{p_{i}}^{2}\times \prod_{j=0}^{l}R_{q_{j}}^{2}$. Based on $\mathrm{SWKGen}(s_{1},s_{2})$ and using a secret key *s*, the relinearization key is generated by denoting $\mathrm{RK}=\mathrm{SWKGen}(s^{2},s)$, and the rotation key for the index *r* is generated as ${\mathrm{RotK}}_{r}=\mathrm{SWKGen}\left(s^{5^{r}},s\right)$. Note that these keys are selectively generated based on the application. For example, if the application only consists of arithmetic operations, ${\mathrm{RotK}}_{i}$ is never used and therefore does not need to be generated. We hereafter focus on the generation of rotation keys, which are dynamically selected based on the rotation indices required by a given application, as relinearization keys exhibit comparatively lower variability. Using the above definition, the rotation keyset structure is determined by three main parameters: the rotation index (*r*), the ciphertext level (*l*), and the number of temporal primes (α). The rotation index (*r*) defines how far the ciphertext slots are shifted. To support all possible rotations, a separate rotation key must be precomputed for each index. However, storing keys for every possible rotation would consume a lot of memory. To reduce memory overhead, widely used FHE libraries [59,60] store rotation keys only for power-of-two indices. When a non-power-of-two rotation is needed (e.g., r=7), it is decomposed into a sequence of power-of-two rotations (e.g., 4 + 2 + 1), requiring multiple operations instead of one. The ciphertext level (*l*) represents how many more multiplications can be performed before bootstrapping is needed, as the ciphertexts are initialized at the highest modulus $Q_{max}$ and gradually decrease through modulus switching as the computations progress. Since key switching depends on the modulus level, each level requires a corresponding key. The number of temporal primes (α) indicates how many primes are appended to split ciphertext coefficients before key switching. Figure 3 shows the available options of α at different multiplication depths in a bootstrappable setup with $N=2^{16}$. For example, for a multiplication depth of 9, α = 5 and α = 1 are two options that developers can choose.

## 3. Motivation

This section introduces two key observations that motivated our research. First, we examine the challenges associated with selecting the appropriate parameters for the rotation keyset design, which significantly impact the efficiency of FHE-based computations. Then, we present a preliminary analysis of the trade-off between memory consumption and computational performance in encrypted neural network inference.

### 3.1. Difficulty in Parameter Setting for Key Switching

Key switching is one of the most computationally expensive operations among RNS-CKKS operations, particularly in ciphertext multiplication and rotation. As shown in Table 2, these operations exhibit significantly higher latency than other FHE primitives due to the key-switching process. Moreover, rotation plays a dominant role in bootstrapping latency, as it is executed multiple times during the bootstrapping process, especially in the coeffToSlot and slotToCoeff transformations. Thus, the efficiency of key switching directly affects the overall performance of an FHE-based application.

Among the various parameters in FHE, α (or dnum) is the main parameter directly affecting the key-switching efficiency. The variables α and dnum are used during the key-switching process: α denotes the number of temporary primes temporarily added to a ciphertext, and dnum represents the number of decomposition digits, which determine the extent to which the ciphertext is expanded. When the maximum multiplicative depth (#Q) is present, α and dnum need to satisfy the condition,

(2) $$\mathrm{dnum}=\left\lceil \frac{\left(1+\#Q\right)}{\alpha }\right\rceil .$$

To simplify the discussion, we consider only cases where (1+#Q) is divisible by α, which aligns with the default setting of the widely used FHE libraries [59,61]. The ciphertext modulus (QP) should be split into two parts: one for $Q=\prod_{i=0}^{\#Q}q_{j}$ and another for temporal primes, $P=\prod_{j=0}^{\alpha -1}p_{j}$. Since the total modulus budget (i.e., the QP pool) is limited to achieve a certain security level (e.g., 128-bit security), developers must carefully allocate it. This allocation directly impacts the performance of RNS-CKKS operations, including bootstrapping and rotation, as well as the total multiplicative depth supported by the circuit. For example, bootstrapping with α=5 is 1.65× faster than with α=1, although the former supports a maximum multiplicative depth of 9 compared to 16 for the latter. Despite its significant impact, it has not been thoroughly analyzed. Prior works [53,62] typically rely on pre-defined parameter settings from existing libraries.

**Figure 4:**
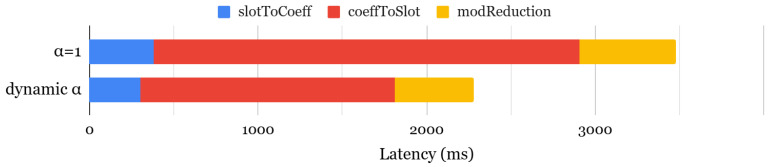
Comparison of bootstrapping latency.

Recent work [63] has proposed a dynamic α selection framework to optimize key-switching efficiency by dynamically selecting the best α configuration at each ciphertext level. Unlike static configurations, this approach evaluates various α options at runtime and chooses the optimal one while preserving the required security level. Figure 4 compares bootstrapping latency between static and dynamic α configurations, showing that the dynamic approach accelerates the slotToCoeff and coeffToSlot operations by 1.27× and 1.67×, respectively, leading to an overall 1.52× speedup. However, this performance gain comes at the cost of increased memory usage, as rotation keys must be generated separately for different α values, resulting in 1.52× higher memory consumption. While the original work focused on polynomial evaluation, we extended this analysis to assess the impact of dynamic α selection on both bootstrapping and encrypted neural network inference, providing a more comprehensive understanding.

### 3.2. Memory–Latency Trade-Off of Rotations in Neural Networks

FHE-based neural networks require frequent ciphertext rotations, particularly in arithmetic layers such as convolutional and fully connected layers. This overhead is further amplified in multiplexed convolution, a technique that maximizes slot utilization by packing multiple data points, including multiple channels, into a single ciphertext. This method employs single-input single-output (SISO) convolution [64], improving ciphertext slot utilization but increasing the number of required rotations. For example, FHE-MP-CNN [54] generates a rotation keyset of 257 indices for CIFAR-10 ResNet-20. To support multiple rotation indices in neural networks, existing approaches typically employ one of two key-generation strategies:

- **All-Required Key Generation**: This approach precomputes rotation keys for all required indices in the neural network. Although it eliminates additional rotations, it demands excessive memory storage, making it impractical for large-scale models.
- **Power-of-Two Key Generation**: Instead of generating keys for all indices, this approach generates rotation keys only for power-of-two indices and performs recursive rotations to achieve arbitrary shifts. Although this method reduces memory consumption, it introduces additional computational latency. For example, a rotation of index 3 requires two consecutive rotations (2 + 1) in the power-of-two approach, whereas the all-required method completes rotation in a single step.

Several widely used FHE libraries, including HEAAN [59] and SEAL [60], implement the power-of-two approach to optimize memory usage. However, this optimization comes at the cost of an increased inference latency due to additional rotations. Conversely, FHE-MP-CNN adopts the all-required strategy to minimize rotation overhead, but this results in excessive memory consumption. In our experiments, we found that storing rotation keys for all required indices in FHE-MP-CNN needs approximately 307 GB, which is 9× larger than the memory footprint of the power-of-two approach. This stark contrast highlights a fundamental trade-off. While aggressive key generation improves computational efficiency, it leads to impractically high memory usage, whereas memory-efficient key-generation strategies increase inference latency due to recursive rotations.

## 4. Related Works

### 4.1. Performance Optimization on FHE and FHE-Based Neural Networks

To mitigate the performance bottlenecks associated with FHE, extensive research efforts have been devoted to accelerating its computations. Some of them introduced hardware accelerator designs [51,52,65,66] that take advantage of the inherent parallelism of FHE operations and efficiently map them to fully utilize the high computational capabilities of various hardware platforms such as FPGA [52], cloud service instances with GPUs [66], and customized ASIC chips [51]. Some others introduced FHE operation schedulers [53,67,68,69,70], micro-scheduling the FHE operation primitives in order to reduce computational latency. Others devised customized sets of FHE operations tweaked for specific applications. For example, FHEMPCNN [54] and HCNN [71] introduced efficient methods for computing convolutional layers using FHE. AutoFHE [55] and AESPA [72] adopt re-training and post-processing to design FHE-friendly neural networks. Although these prior works have contributed significantly to improving FHE efficiency, they have primarily focused on optimizing computation itself. However, a critical aspect that has been largely overlooked is the design of the *FHE keyset*, which plays a crucial role in balancing computational efficiency and memory consumption. In this work, we focus on the FHE keyset and propose a method that selectively generates only the necessary keys by considering not only rotation indices but also ciphertext levels, thereby improving overall system efficiency.

### 4.2. Keyset Design Space Exploration

Several prior works have investigated the potential of extending keysets to improve performance. Two studies [63,73] explored increasing dnum to enhance key-switching efficiency. These works introduced methodologies to ensure that the keyset configuration achieves optimal latency for single multiplications. Other studies [61,74,75] focused on generating extended rotation keys to accelerate ciphertext rotations. However, these approaches fail to explore the full keyset design space, as they do not account for all three keyset components simultaneously. Our work differs in that it systematically explores the entire keyset design space by considering all three factors—α selection, rotation index selection, and ciphertext-level optimization—as interdependent variables. Doing so provides a more comprehensive framework for optimizing keyset configurations in homomorphic encryption.

### 4.3. Keyset Design in Other Homomorphic Encryption Schemes

Although our study focuses on the CKKS scheme, we note that our findings are applicable to other Ring Learning With Error (RLWE)-based schemes such as BGV and BFV. These schemes share similar structural properties with CKKS, suggesting that our keyset optimization approach can also be extended to optimize their performance. On the other hand, FHEW and TFHE rely on Learning With Errors (LWEs), which exhibits fundamentally different characteristics. Unlike CKKS, these schemes execute operations in a long sequential loop, where each component of the secret key is computed in order. Despite this difference, they still rely on keyset configurations, albeit with different key size and structure determinants. We anticipate that similar research could be conducted for these schemes to explore keyset optimization strategies tailored to LWE-based cryptosystems, which we leave as a future research direction.

## 5. Keyset Design Space (KDS) Exploration

In this section, we analyze the impact of three key factors that influence the efficiency of key switching: α (or dnum), rotation index, and ciphertext level. To ensure that our analysis focuses solely on key switching, we fix the bootstrapping parameters and set the scaling factor to Δ = 2^50^. For a bootstrappable FHE setting with N = 2^16^, the bootstrapping procedure consumes 15 levels. In this configuration, the maximum multiplicative depth of a ciphertext is 31 when α = 1, including bootstrapping.

### 5.1. Impact of α

**Figure 5:**
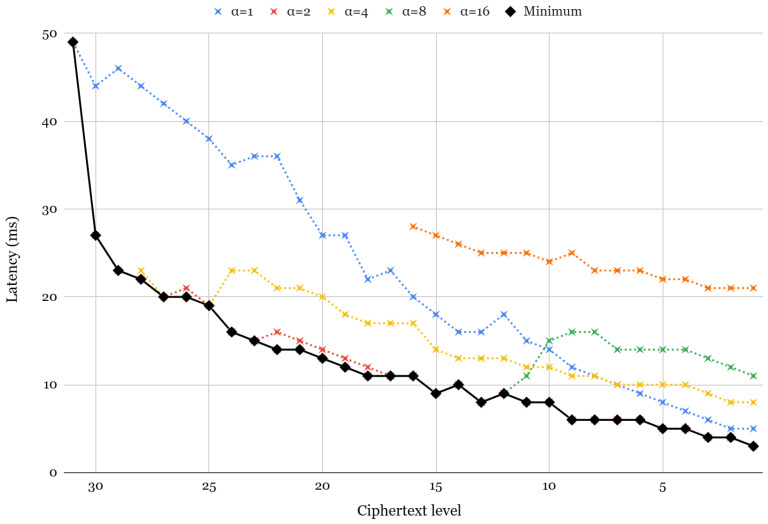
Latency of relinearization for different α values.

To evaluate the impact of α on key-switching performance, we first measure the latency of ciphertext multiplication (with relinearization) on different α values, as shown in Figure 5. This analysis follows the framework proposed in [63] and provides insight into how varying α influences key-switching efficiency. Note that since all ciphertext must satisfy 128-bit security during key switching, the starting level to apply α varies. Each dotted line in the figure represents the rotation latency for static α values of 1, 2, 4, 8, and 16, whereas the solid line indicates the optimal α selection at each level. Key switching involves a transformation known as modUp, where the ciphertext is expanded to a higher modulus before performing operations with the switching key. The effective size of the ciphertext during key switching at level *l* is given by the following:

(3) ciphertext size=(1+l)+α.

Thus, for lower ciphertext levels, increasing α does not always improve latency, as additional polynomials introduced by modUp can create computational overhead.

**Figure 6:**
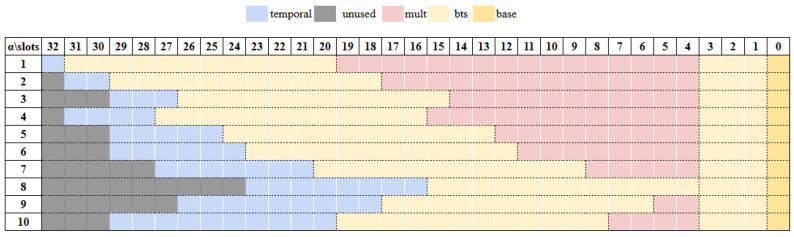
Grid of prime usage.

Moreover, the impact of α varies depending on whether bootstrapping is used. In this study, we fix the bootstrapping configuration (e.g., approximated function for the modReduction phase) to isolate the impact of α selection. During bootstrapping, ciphertexts are first transformed in slotToCoeff; then, their modulus is raised to the highest level, followed by a sequence of modReduction. Figure 6 provides a visualization of the prime usage in different values of α, optimized for the maximum multiplication depth. Each α configuration determines a unique multiplicative depth, and the gray slots in the figure represent unused primes that are excluded from the total modulus count, providing a clearer view of the α allocation. The yellow slots indicate primes dedicated to bootstrapping, which consumes 15 levels. For a 128-bit security setting, a ciphertext achieves a maximum multiplicative depth of 31 when using a single temporal prime (α = 1), which allows up to 16 sequential multiplications. As α increases, the available multiplicative depth generally decreases; however, due to the inverse relationship between dnum and α, this dependency leads to non-uniform trade-offs between α selection and computational efficiency.

**Figure 7:**
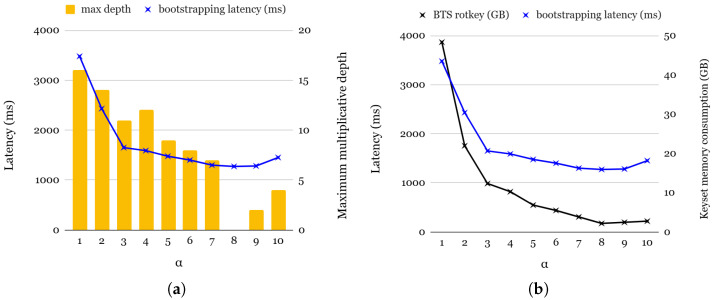
(**a**): Bootstrapping latency and rotation keyset memory consumption for bootstrapping; (**b**): bootstrapping latency and maximum depth for multiplication.

To further analyze the impact of α, we measure both the bootstrapping latency and the memory consumption of the rotation keysets. Figure 7a presents the bootstrapping latency and maximum multiplication depth across different α values. Since bootstrapping involves multiple key switchings, including ciphertext rotations in coeffToSlot and slotToCoeff, as well as Cmult operations in modReduction, the choice of α significantly influences the overall latency. Lower α values correspond to higher starting levels in the coeffToSlot step, resulting in slower bootstrapping. Although higher α values generally improve bootstrapping performance, we observe an anomalous slowdown for α = 9 and α = 10. This is attributed to the ciphertext’s expansion caused by modUp, which offsets the expected benefits of higher α values. For practical applications requiring various multiplication depths, selecting an optimal α is essential. Among the range of viable α values (3 to 7), all exhibit similar bootstrapping latency. However, α = 4 provides the best trade-off by achieving the highest multiplicative depth while maintaining a reasonable bootstrapping efficiency.

Figure 7b presents the memory consumption of the rotation keyset during bootstrapping. Interestingly, memory consumption decreases as α increases. The total memory footprint of the rotation keyset at level *l* is determined by the following:

(4) 2×N×dnum×(1+l+α)×(# of required indices)×(data size of coefficient).

Since α and dnum are inversely proportional, lower α values typically result in lower memory consumption. However, there are exceptions for α = 9 and α = 10, where an increase in #Q leads to slightly higher memory usage compared to the case of α = 8.

### 5.2. Impact of Rotation Index Selection

To generate a rotation keyset for many rotations in neural networks, two main approaches have been proposed: all-required and power-of-two. The all-required approach generates all necessary rotation keys by performing a pre-analysis of the neural networks’ architecture to identify the required rotation indices. This allows all rotation calls in the neural network to be handled in a single rotation call, minimizing rotation latency. On the other hand, the power-of-two approach generates keys for indices that are a power of two, including both positive and negative values. It then performs recursive rotation calls using these generated keys, which introduces additional computational overhead, as shown in Table 3. For example, a rotation index *k* with a hamming weight of 3 is approximately 3.8× slower than a rotation index of 1.

This difference also affects the overall latency of neural network inferences. Table 4 shows the latency of CIFAR-10 ResNet-20 using two different types of ReLU evaluations. Since MPCNN uses a higher degree of ReLU compared to AESPA, it consumes more levels, resulting in greater multiplication depth and more frequent bootstrapping. For the rotations in each neural network evaluation result, the all-required approach achieves faster latency compared to the power-of-two approach: 1.95× for MPCNN and 1.93× for AESPA, respectively. The other operations and bootstrapping are not affected by the index selection approach. As a result, the overall latency of the all-required approach is 1.14–1.36× faster than the power-of-two approach, thanks to the non-recursive rotation calls. However, there is also a disadvantage, which is the increase in memory usage for the rotation keyset. Although the power-of-two approach generates rotation keys for the 2×log(N/2)−1 indices, the all-required approach should generate additional keys for all required, necessary indices. This typically exceeds the number of rotation keys in a keyset generated by power-of-two (e.g., 257 indices are required for ResNet-20), resulting in higher memory consumption.

### 5.3. Impact of the Ciphertext Level

**Figure 8:**
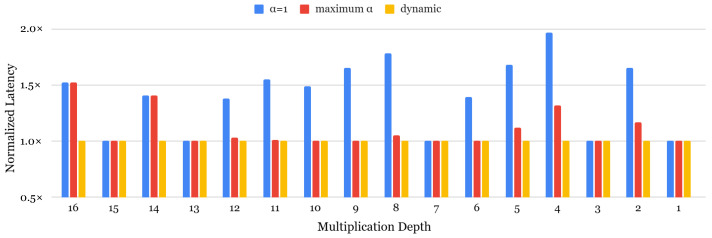
Bootstrapping speed up of dynamic α compared to static α.

The ciphertext level is a new factor when considering a rotation keyset, which has not been the main focus of previous work. Our key observation is that ciphertext levels for rotations are often set lower than the maximum level, even though rotation keys are typically generated up to the maximum level by default in FHE libraries. This implies that rotation keys for higher levels are never used, introducing unnecessary memory waste. Note that this consideration of levels does not significantly impact latency in our evaluation environment. Therefore, we aim to optimize this unused memory allocation to minimize overall memory consumption. To advance our dynamic α selection approach with level-aware rotation keyset generation, we first analyze the impact of bootstrapping latency in the dynamic α approach [63], as shown in Figure 8. For almost all multiplication depths, the dynamic α selection strategy achieves the fastest latency. Since the smaller multiplication depths allow more α to be used at higher levels, the acceleration effect is particularly evident compared to α = 1. There are two special cases for the effects of dynamic α. For one special case, for multiplication depths with 15, 13, 7, 3, and 1 values, the dynamic α strategy does not show a noticeable acceleration effect compared to α = 1 due to the fact that (1+#Q) is a prime, where the only available α is 1 for these depths. Similarly, for multiplication depths of 9 and 6, the latency results for dynamic α and max α are identical. This is because, when evaluating all possible α values for each depth, with {1, 5} for depth 9 and {1, 2} for depth 6, the ciphertexts at each level are optimized to α = 5 and α = 2, respectively. As a higher α is not beneficial for low-level ciphertexts, a dynamic α achieves the fastest performance in all cases, averaging 1.41× in speed-up value compared to α = 1 and 1.12× in maximized α cases.

To reduce memory consumption, we propose a level-aware rotation keyset generation strategy, which only generates the keys that are actually needed considering the ciphertext level, including the rotation keyset for bootstrapping. The rotation keyset is generally generated from level 0 to the maximum available level (1 + #Q), as defined by the parameter setting. This also generates rotation keys that will never be used, inducing memory waste. Table 5 describes the total memory consumption of the rotation keyset for bootstrapping. As mentioned earlier, for multiplication depths of 15, 13, 7, 3, and 1, dynamic α does not provide a benefit, since the only available α option is 1. For other cases, dynamic α results in lower memory consumption compared to the α = 1 case. However, compared to the max α approach, pure dynamic α uses relatively larger memory because it dynamically generates keys for multiple α values. To optimize this, we generated a rotation keyset in a level-aware manner, which is noted as dynamic+level-aware in the table. In some cases, the memory usage of our strategy is greater than the max α approach, but this trade-off enables a comparable level of performance improvement, as shown in Figure 8.

### 5.4. Combining the Three Factors to Generate a Rotation Keyset for Neural Networks

**Figure 9:**
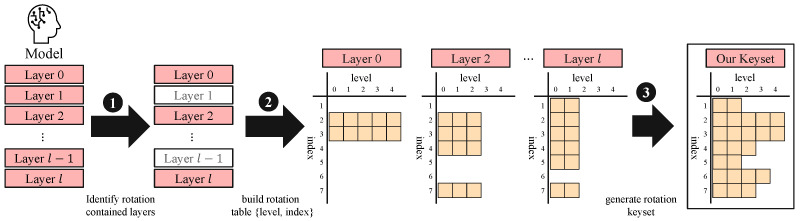
Overview of our approach.

Combining insights from the three factors of KDS presented above, we extend our level-aware strategy to neural networks, as demonstrated in Figure 9. We begin by analyzing the neural network’s computation to identify the specific level status of rotation calls at certain layers, determining which levels are actually used. Since our goal is to generate the rotation keyset considering the neural network, we exclude layers such as activation or batch normalization, which operate in an SIMD manner and do not perform rotations. Then, we construct a rotation table for each layer that involves rotations and mapping rotation indices against ciphertext levels. This provides a comprehensive view of rotation requirements across the entire neural network. By doing so, we can construct a minimal set of required rotation keys for the target neural network. Finally, we generate level-aware rotation keys based on the rotation table with optimized α for each level. This approach ensures compatibility with the RNS structure while avoiding the generation of unnecessary keys. By precisely tailoring the key generation to the actual needs of the neural network, we achieve substantial memory savings without sacrificing the ability to perform any required rotation.

## 6. Evaluation

### 6.1. Experimental Setup

We conducted our experiments on a system equipped with two Intel Xeon Gold 6326 CPUs (2.9 GHz), providing 64 threads and 1TB of main memory. All evaluations were performed on Ubuntu 22.04 LTS using the HEaaN library [59]. We measured the memory consumption of rotation keys used for inference and bootstrapping to evaluate the trade-offs between latency and memory efficiency. To demonstrate the effectiveness of our approach in real-world scenarios, we present two case studies with experimental results.

### 6.2. Compared Baselines

We compared our method with several baseline approaches based on different keyset design space (KDS) factors, including index selection, α selection, and level selection.

- **Index Selection Baselines:** We considered two widely adopted index selection strategies:
  – **Power-of-Two** (Pot): Generates a default rotation keyset consisting of power-of-two indices (both positive and negative) and performs recursive rotations for arbitrary indices.
  – **All-Required**: Generates rotation keys for all indices required during inference, eliminating the need for recursive rotation calls.
  The keyset generated by all-required depends on the benchmark, whereas power-of-two produces the same keyset across all benchmarks.
- **α Selection Baselines:** We compared our approach with two different α configurations:
  – **α = 1** (P1): A standard option in several FHE libraries [60], which maximizes the available multiplicative depth.
  – **Dynamic α** (Pdyn): A strategy that dynamically selects the optimal α to minimize key-switching latency.
- **Level-Selection Baselines:** We compared our approach with a naive full-level key-generation strategy, where rotation keys are generated for all ciphertext levels. Since prior research has not explicitly considered level-aware key selection, this serves as a reference for evaluating the effectiveness of our approach.

### 6.3. Case Study 1: Wireless Intrusion Detection System (IDS) Based on Gated Recurrent Unit (GRU)

This intrusion detection system (IDS) case involves a wireless sensor that captures network traffic and analyzes it to determine whether the activity is malicious. As identified in previous works [76,77,78], attributes extracted from network traffic can reveal sensitive internal device behaviors, such as root privileges or user actions. Although a single packet may not appear sensitive on its own, it can indirectly expose private information, for example, by inferring the presence of a specific individual [79]. For our evaluation of IDS, we use the NSL-KDD dataset [38], an improved version of the widely used KDD99 [37] dataset. Each record in NSL-KDD contains 41 features and is labeled in one of five categories: benign or one of four attack types—Denial-of-Service (DoS), User-to-Root (U2R), Remote-to-Local (R2L), and Probing (Probe) attacks. As our baseline model, we adopt a GRU-based model proposed in prior work [39], demonstrating a high attack classification accuracy of 99.19%. We followed the data pre-processing method and used the same model architecture proposed in the original paper. The model consists of one GRU layer with 128 hidden units followed by three fully connected layers using the sigmoid activation function. To evaluate non-arithmetic functions under FHE, we use the degree-7 polynomial approximation implemented in MHEGRU [80].

#### 6.3.1. End-to-End Inference Latency

**Figure 10:**
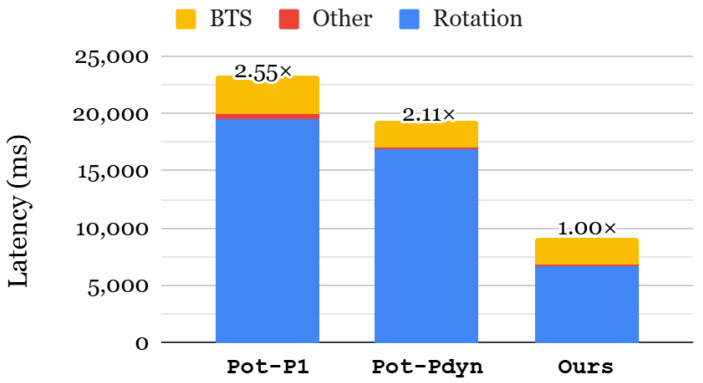
End-to-end latency of GRU-MLP using NSL-KDD dataset.

Figure 10 shows the end-to-end latency of the GRU-MLP model, comparing different KDS approaches. Since the latency of our method is identical to the all-required for index selection, we omit its result. The most time-dominant part of the GRU-MLP model is rotation because both GRU and MLP layers rely heavily on matrix–vector multiplications, which involve numerous rotations. We first evaluate the impact of dynamic α selection by comparing Pot-P1 and Pot-Pdyn. Dynamic α selection reduces rotation and BTS cost by 1.16× and 1.52×, respectively, resulting in an overall inference speedup of 1.21×. Since the GRU-MLP model requires 254 distinct rotation indices, the all-required index selection results in a 2.53× speedup in rotation, leading to a 2.11× improvement in the end-to-end inference latency.

#### 6.3.2. Memory Consumption

Table 6 presents the memory consumption of the rotation keyset for the GRU+MLP model. As previously discussed, the model requires 254 distinct rotation indices, resulting in huge memory overhead with the all-required index selection. In contrast, the power-of-two index selection substantially reduces the memory requirement by 5.50–6.61× compared to all-required in different α configurations. However, as shown in Figure 10, power-of-two selection is 2.11–2.55× slower than all-required. To retain the performance benefits of all-required while mitigating its memory overhead, we adopted a level-selection strategy that only generates the necessary keys. Since dynamic α selection includes many unused keys at different levels, our selective key-generation approach efficiently reduces the overall memory usage. As a result, our proposed KDS improves both latency and memory efficiency over prior methods.

### 6.4. Case Study 2: Image Classification Based on Convolutional Neural Network (CNN)

Convolutional neural networks (CNNs) are widely adopted for analyzing image data, making them well-suited for sensors such as cameras that produce image data. To assess the effectiveness of our approach on CNNs, we evaluated two widely used architectures—ResNet-20 [6] and SqueezeNet [81]—on the CIFAR-10 dataset. Additionally, we compared our results with two baseline FHE CNNs that differ in ReLU depth: one using a low-depth ReLU function (AESPA) and another employing a high-depth ReLU function (MPCNN). AESPA utilizes a square function as its activation function, which consumes only one level, whereas MPCNN employs a composite function composed of multiple polynomials [82], resulting in significantly deeper multiplicative depths (e.g., 14).

#### 6.4.1. End-to-End Inference Latency

**Figure 11:**
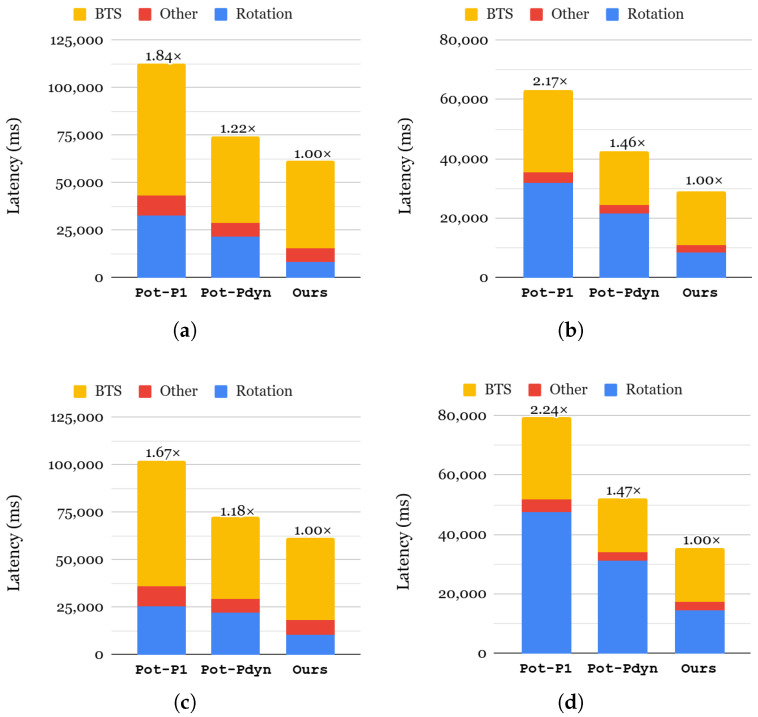
Comparison of the end-to-end latency of neural networks using the CIFAR-10 dataset (**a**): SqueezeNet using MPCNN; (**b**): SqueezeNet using AESPA; (**c**): ResNet-20 using MPCNN; (**d**): ResNet-20 using AESPA.

Figure 11 presents the end-to-end inference latency results for our approach compared to two different α selection strategies. Note that level selection does not impact inference latency but rather memory consumption. Therefore, we do not provide an evaluation result of the latency between different level-selection approaches. For two approaches, Pot-P1 and Pot-Pdyn, we chose power-of-two as their index selection to highlight the effectiveness of each KDS factor sequentially. Compared to Pot-P1, Pot-Pdyn achieves 1.41–1.52× faster inference for each neural network evaluation thanks to the acceleration in key switching. As represented in each bar, the latencies of all components are reduced, especially by 1.16–1.52× in rotation and 1.49× in bootstrapping. This demonstrates the effectiveness of Pot-Pdyn selection compared to Pot-P1 for neural networks. The difference between our approach and the Pot-Pdyn approach lies in index selection, and we use all-required. By generating all indices required for a network, our approach improves the rotation latency, which in turn enhances the performance of overall inferences: 1.22–1.45× for SqueezeNet and 1.18–1.47× for ResNet-20.

#### 6.4.2. Memory Consumption

Table 7 summarizes the memory consumption of rotation keysets under different KDS factor selections. The all-required index selection requires the highest memory overhead of over 490 GBs as it generates all indices that are needed, resulting in greater memory consumption compared to the power-of-two index selection. For α selection, the dynamic α strategy nearly doubles memory consumption because it requires storing multiple sets of rotation keys for different α values. While this configuration improves computational efficiency, it introduces a substantial trade-off in terms of storage requirements. The last row of Table 7 presents the results for our approach, which achieves minimal memory consumption compared to all other configurations. Our rotation keyset is carefully designed to generate only the necessary keys up to the required ciphertext level for each neural network. For example, instead of generating rotation keys up to the maximum multiplicative depth, we limit key generation to the highest level required after bootstrapping (e.g., level 19 for a multiplication depth of 16). This significantly reduces memory usage while maintaining computational efficiency. Another notable finding is that our method is the only one that generates distinct keysets for all four benchmark cases, demonstrating its adaptability to different neural network architectures. Unlike baseline approaches that apply the same keyset to all benchmarks, our strategy dynamically adjusts key selection based on application-specific requirements, further optimizing memory efficiency.

## 7. Discussion and Future Work

### 7.1. Deploying Neural Networks Directly on IoT Devices

The simplest way to protect a user’s private data is to perform inference locally on the IoT device itself. To accommodate local inferences on IoT devices, several works have sought wisdom from quantization or pruning [83,84,85,86], which made it possible to efficiently deploy AI models on edge devices by reducing the computational and memory overhead [8,87,88]. However, such approaches are not one-way optimizations but induce trade-offs with accuracy, degrading the quality of the model. The impact is frequently severe, having an accuracy drop by up to 60% [83]. To deliver high-quality models without compromise (i.e., without the accuracy loss associated with quantization), AI service providers typically perform inference on remote servers through MLaaS, which is the scenario adopted in this work. Additionally, from the perspective of service providers, who consider their trained models intellectual property, it is possible to avoid disclosing the model to clients.

### 7.2. Application to Diverse Neural Networks

Numerous studies have explored efficient methods for evaluating neural networks over FHE, including AESPA and MPCNN. For instance, AutoFHE [55] dynamically assigns polynomial approximations to different ReLU layers within a convolutional neural network (CNN), using a neural architecture search to predict their impact on accuracy and performance. In our evaluation on the AESPA model, which adopts the lowest-degree polynomial as its activation function, we observed the most varied distribution of ciphertext levels across layers. Based on this observation, we envision that our approach can effectively support the dynamic selection of activation functions. In addition, our approach is broadly applicable to a wide range of FHE-based neural networks, as it does not impose limitations on analyzing rotation indices. By only generating the necessary rotation keys while accounting for ciphertext levels, we anticipate that our method can improve efficiency across different architectures.

### 7.3. Future Works

In this subsection, we outline several directions for future work to help the research community further advance this study:

- **Automated FHE Keyset Configuration:** The automation of FHE-applied applications has been widely studied in recent years. Several works have aimed to improve usability by automating various aspects of FHE deployment, such as FHE parameter selection [67], bootstrapping placement [53,55], and FHE-specific operation scheduling (e.g., rescaling) [89,90]. Building on these efforts, developing an automated algorithm to determine the optimal FHE keyset configuration represents a promising research direction. Given our observation that optimized keysets can lead to significant improvements in either memory efficiency or inference performance, designing keyset selection algorithms tailored to application-level requirements is a valuable next step.
- **FHE Keyset Configuration via Key Extension:** While our work focuses on dynamic α selection, a recent study [73] proposed extending key sizes to improve performance. Specifically, they decompose keys for certain operations (including rotations), which increases memory overhead but reduces the complexity of NTT/iNTT transformations. Although this approach shows promise, several challenges remain before it can be practically implemented, including managing the trade-off between memory usage and execution speed, as well as integration with existing FHE toolchains. First, extending the key obviously induces much higher memory consumption, so performance will highly depend on the memory bandwidth of the running hardware platform. Considering that the minimal keyset already consumed tens of GB, the increase leads to higher memory transfer latency. Meanwhile, note that the results we acquired are from CPUs with DDR4 DRAM memory, where the memory bandwidth is merely 64 bits. Since memory bandwidth is already low and each key is worth MBs (thus, not many cache hits), memory transfer is already at the peak, requiring reading keys per FHE operations. As a result, the increased memory consumption from the key extension does not lead to extensive performance degradation. The contrast between the impacts suggests that we need to consider the characteristics of the running hardware platform in order to choose the appropriate keyset design because an efficient keyset for a platform does not necessarily lead to good performance on others.

## 8. Conclusions

In this work, we presented a comprehensive analysis of the key design space and introduced an approach to designing an efficient keyset for FHE-based neural networks. We presented three components of the key design space (KDS) and analyzed the impact of each on latency and memory consumption in neural networks. To demonstrate the applicability of our approach to sensor-based devices, we conducted two case studies: one for intrusion detection systems and the other for image classification. Our evaluation demonstrated that our level-aware approach reduces memory consumption by up to 11.29× while achieving an average 2.09× faster latency across different neural networks. We envision that our approach facilitates the practical deployment of FHE-based neural networks for resource-constrained environments, making PPML more feasible in real-world applications.

## Figures and Tables

**Table 1:** Examples of AI models in the literature using datasets collected via sensors.

Type of Sensor	Acquired Data	Dataset	Usagex
Camera	Face	CelebA [11]	Emotion recognition [12] Face edit [13] Image generation [14]
		LFWA [15]	Face recognition [16] Face swapping [17] Image synthesis [18] Image restoration [19]
	Human action	MPII Human pose [20]	Human tracking [21] Pose estimation [22]
		IEMOCAP [23]	Emotion recognition [24]
Bio-sensor	Electrocardiography (ECG)	MIT-BIH [25]	Arrhythmia detection [26,27]
		PTB-XL [28]	Arrhythmia classification [29] Heart disease classification [30]
Accelerometer and Gyroscope	Linear acceleration & Angular velocity	PAMAP2 [31]	Human activity recognition [32,33]
		ADL [34]	Human activity recognition [35,36]
Wireless	Packet	KDD99 [37], NSL-KDD [38]	Intrusion detection system [39,40]

**Table 2:** Latency of RNS-CKKS operations for N = 2^16^ (ms, α = 1).

Level (ms)	4	8	12	16	20	24	28
Padd	3.865	6.187	8.39	10.532	13.054	15.68	18.876
Cadd	1.647	1.671	1.703	1.793	1.515	2.206	2.606
Pmult	4.217	4.219	4.214	5.038	2.938	4.477	4.685
Cmult	86.499	126.161	171.98	235.959	243.882	358.755	462.816
Rescale	44.681	54.322	63.343	68.839	87.189	106.11	118.971
Rotation	73.299	108.481	149.512	193.197	206.114	301.402	415.616
Bootstrapping	3335						

**Table 3:** Relative latency of rotations for N = 2^16^.

Hamming weight	1	2	3	4	5	6	7
Relative latency	1.0	2.6	3.8	5.3	5.8	7.6	8.3

**Table 4:** Comparison of CIFAR-10 ResNet-20 inference latency.

Latency (ms)	ResNet-20 MPCNN		ResNet-20 AESPA	
	Power-of-Two	All-Required	Power-of-Two	All-Required
Rotation	25,264	12,919	47,507	24,637
Other	10,823	10,823	4210	6210
BTS	66,082	66,082	27,824	27,824
Total	102,169	89,824	79,541	58,671

**Table 5:** Rotation keyset memory consumption for bootstrapping.

Multiplication Depth	Static		Dynamic	Dynamic +Level-Aware
	α = 1	Maximum α		
16	40.802 GB	48.318 GB	61.641 GB	25.535 GB
^†^ 15	38.292 GB			
14	35.861 GB	35.861 GB	56.741 GB	18.895 GB
^†^ 13	33.510 GB			
12	31.239 GB	10.239 GB	56.063 GB	10.305 GB
11	29.048 GB	12.316 GB	10.400 GB	10.273 GB
10	26.936 GB	16.562 GB	13.986 GB	13.413 GB
9	24.904 GB	6.842 GB	5.778 GB	5.557 GB
8	22.951 GB	5.474 GB	24.864 GB	6.250 GB
^†^ 7	21.078 GB			
6	19.285 GB	11.938 GB	17.094 GB	9.597 GB
5	17.572 GB	3.822 GB	9.643 GB	4.448 GB
4	15.938 GB	2.737 GB	35.024 GB	4.452 GB
^†^ 3	14.384 GB			
2	12.910 GB	2.454 GB	29.327 GB	4.05 GB
^†^ 1	11.515 GB			

^†^: α can only be 1.

**Table 6:** Memory consumption for rotation keyset of GRU+MLP model.

	Key Design Space (KDS)		GRU+MLP
Index	*α*	Level	
All-required	dynamic	All	553.648 GB
All-required	*α* = 1	All	307.09 GB
Power-of-two	dynamic	All	83.752 GB
Power-of-two	*α* = 1	All	55.835 GB
All-required	dynamic	Needed	52.026 GB

**Table 7:** Comparison of rotation keyset memory consumption.

Key Design Space (KDS)			SqueezeNet		ResNet-20	
Index	*α*	Level	MPCNN	AESPA	MPCNN	AESPA
All-required	Dynamic	All	490.968 GB		534.656 GB	
All-required	*α* = 1	All	253.403 GB		275.952 GB	
Power-of-two	Dynamic	All	108.179 GB		108.179 GB	
Power-of-two	*α* = 1	All	55.835 GB		55.835 GB	
All-required	dynamic	Needed	45.353 GB	46.016 GB	47.32 GB	53.511 GB

## Data Availability

Data are contained within the article.

## Abbreviations

The following abbreviations are used in this manuscript:

IoT	Internet of Things
MLaaS	Machine Learning as a Service
PPML	Privacy-Preserving Machine Learning
RLWE	Ring Learning with Error
FHE	Fully Homomorphic Encryption
KDS	Keyset design space
RNS	Residue Number System
IDS	Intrusion detection system
